# Familial associations between autoimmune hepatitis and primary biliary cholangitis and other autoimmune diseases

**DOI:** 10.1371/journal.pone.0240794

**Published:** 2020-10-20

**Authors:** Hauke Thomsen, Xinjun Li, Kristina Sundquist, Jan Sundquist, Asta Försti, Kari Hemminki

**Affiliations:** 1 Division of Molecular Genetic Epidemiology, German Cancer Research Centre (DKFZ), Heidelberg, Germany; 2 Center for Primary Health Care Research, Lund University, Malmö, Sweden; 3 GeneWerk GmbH, Heidelberg, Germany; 4 Department of Family Medicine and Community Health, Department of Population Health Science and Policy, Icahn School of Medicine at Mount Sinai, New York, New York, United States of America; 5 Center for Community-based Healthcare Research and Education (CoHRE), Department of Functional Pathology, School of Medicine, Shimane University, Matsue, Japan; 6 Hopp Children's Cancer Center (KiTZ), Heidelberg, Germany; 7 Division of Pediatric Neurooncology, German Cancer Research Center (DKFZ), German Cancer Consortium (DKTK), Heidelberg, Germany; 8 Division of Cancer Epidemiology, German Cancer Research Centre (DKFZ), Heidelberg, Germany; 9 Faculty of Medicine and Biomedical Center in Pilsen, Charles University in Prague, Pilsen, Czech Republic; Texas A&M University, UNITED STATES

## Abstract

Autoimmune hepatitis (AH) and primary biliary cirrhosis (PBC) are autoimmune diseases (AIDs) targeting cellular components of the liver. Being rare diseases, limited data are available about familial risks among these AIDs (concordant) or between them and other AIDs (discordant). We aimed to carry out an unbiased study on these AIDs based on medically diagnosed patients.

We collected data on patients diagnosed in Swedish hospitals with AH, PBC and other AIDs and calculated familial standardized incidence ratios (SIRs) for concordant and discordant familial relative risks. The number of AH patients was 6,269, of whom 43.0% were male; patient numbers for PBC were 4,269, with 17.8% males. AH accounted for 0.8% and 0.6% of all hospitalized AIDs in Sweden. For AH only the familial risk between siblings was significant (3.83). For PBC the risks for offspring of parents (9.05) and siblings (10.88) were high, but only risk for females was significant. Spousal risks were very high, 5.91 and 6.07 for AH. Risk for AH was 2.21 in families of PBC, and it was 2.47 for PBC in families of AH patients. Among other AIDs, 14 showed a significant association with AH, compared to 16 AIDs with PBC. The surprising finding in this nation-wide family study on medically diagnosed patients was the high risk for AH (6.0) between spouses, which exceed the risk between siblings, suggesting the existence of strong environmental risk factors. AH and PBC were associated with multiple other AIDs. The results call attention to environmental factors in AID etiology which should also be in focus in taking anamnestic data from patients.

## Introduction

Autoimmune hepatitis (AH, formerly known as lupoid hepatitis) and primary biliary cholangitis (PBC, formerly known as primary biliary cirrhosis) are autoimmune diseases (AIDs) of the liver [1]. The incidence of AH was 2/100,000 in UK until year 2015 and a 10-year cumulative mortality was 32% of which liver-related mortality, including hepatocellular carcinoma, accounted for a third [2]. In Sweden the incidence has been estimated at 0.85/100,000 [3]. Both of these incidence rates are close to the internationally reported rates ranging from 0.9 to 5.8/100,000 [4]. The incidence of PBC was 2.6/100,000 in Sweden until year 2014 and a 10-year mortality was 63% for men and 41% for women [5]. Both AIDs have typical clinical presentation and histologic features: AH shows prominent portal and lobular lymphoplasmacytic inflammation while PBC shows chronic progressive destruction of small intrahepatic bile ducts with portal inflammation and ultimate fibrosis [1, 6]. Both are characterized by a T-cell or other immune cell-mediated response to liver self-antigens in genetically predisposed individuals [7]. The two diseases may occur also in the same individuals [5]. AH shows elevated aspartate transaminase and alanine transaminase levels, presence of autoantibodies and increased immunoglobulin G levels [8]. The serological hallmarks of PBC are the presence of anti-mitochondrial autoantibodies (AMA) and a high serum level of immunoglobulin M [6]. The clinical presentation may vary substantially, ranging from an asymptomatic elevation of liver enzymes to acute liver failure. Diagnostic guidelines of the European Association for the Study of the Liver (EASL) are applied in Sweden (5). First-line therapy is immunosuppression for AH and ursodeoxycholic acid for PBC; in advance cases liver transplantation may be required [1, 9].

AH and PBC are typically complex diseases with heterogeneous etiology. Two types of AH have been identified; the more common type 1 is positive for anti-nuclear and/or anti-smooth muscle antibodies while AH type 2 is positive for anti-liver kidney microsomal antibodies and/or anti-liver cytosol type 1 antibody [10]. The existence of molecular mimicry has been confirmed for AH type 2 where the immune response is directed to the liver enzyme cytochrome P450 2D6: this cytochrome shows homology with proteins present in patients with infections caused by hepatitis C virus and herpes viruses [10]. In PBC the mechanisms by AMA and other antibodies produced liver tissue injury remain unknown, however, exposure to infectious pathogens, medication or other compounds are suggested to initiate an immune reaction with participation of innate immunity at the primary stage of PBC [6, 11]. Molecular mimicry is also known for PBC through viral and bacterial peptides that are structurally similar to AMA pathway related epitopes [6, 11]. Both AH and PBC patients may present with polyautoimmunity, i.e., same patients presenting with other AIDs, such as Sjogren’s syndrome, Hashimoto’s thyroiditis, rheumatoid arthritis, sarcoidosis, discoid and systemic lupus erythematosus, Crohn’s disease, ulcerative colitis and celiac disease [5, 11, 12]. Although family members of AH patients had an increased risk of AH (relative risk 4.9) they did not have other AIDs [12, 13]. For PBC, familial relative risk between siblings have been reported at about 10 based on relatively old interview studies [14]. More recently data from the Icelandic genealogical database was used to assess familial relations in first-, second- and third-degree relatives of the PBC patients: the results were consistent with heritable background with relative risks ranging from 9.13 to 3.61 and to 2.59, respectively [15]. Genome-wide association studies have identified human leukocyte antigen (HLA) and non-HLA alleles that are associated with PBC [14]. However, these alleles are non-PBC specific and most of the identified non-HLA loci were also found to be susceptible genes in other AIDs [16].

Due to the sparsity of family studies in medically diagnosed AH and PBC, we used the Swedish hospital data to characterize concordant (for the same AID) and discordant (for different AIDs) diseases. The discordant analysis was conducted bidirectionally, first the relative risk for any other AID in families of AH or PBC, and second the relative risk of AH or PBC in families of other AIDs. Also spousal risks were assessed to examine the potential influence of shared environmental factors.

## Methods

In August 2020 AID patients were identified from the Swedish Hospital Discharge Register (years 1964 through 2012, full national coverage from 1986 onwards) and the Outpatient Register (2001 through 2012). Only the first AID diagnosis was included. Of a total of 769,991 patients with any of 43 AIDs were identified, 51% from the Inpatient Register and 49% from the Outpatient Register. Various revisions of the International Classification of Diseases (ICD) codes were used for identification of AIDs as described elsewhere 24. Family relationships were obtained from the Multigeneration Register, containing the Swedish population in families and covering parental generations for a century 23. As family members, only first-degree relatives of offspring-parent pairs and siblings were considered; ‘the offspring generation’ was born after 1931 and siblings could be defined only in this generation; ‘the parental generation’ was born any time earlier. By year 2012, the offspring generation reached age 80 years. For the parental generation there was no age limits. Information from the registers was linked at the individual level via the national 10-digit civic registration number. In the linked dataset, civic registration numbers were replaced with serial numbers to ensure the anonymity of all individuals. Spouses were identified through the first common child.

Standardized incidence ratios (SIRs) were calculated for the offspring generation as the ratio of observed to expected number of cases. The expected numbers were calculated for all individuals in the offspring generation without a first-degree family history of a specific AID, and the rates were standardized by 5-year-age, gender, period (5 years group), socioeconomic status (six groups: blue-collar worker, white-collar worker, farmer, self-employed, professional, or other/unspecified) and residential area (three groups: large cities, South Sweden, North Sweden). The 95% confidence interval (95%CI) of the SIR was calculated assuming a Poisson distribution. Separate SIRs were calculated for offspring when only parents or only siblings were probands, i.e., they were diagnosed with concordant AID. In analysis of discordant AIDs bidirectional (i.e., AH-AIDx and AIDx-AH) associations were considered, and the methods were as above.

### Ethics

The guidelines of the Helsinki Declaration were followed. The study was approved by the Regional Ethical Review Board in Lund. The permission included use of data from the Swedish Hospital Discharge Register, Outpatient Register and Multi-generational Register. All data were fully anonymized, and at no stage had the authors access to identifiable individuals.

## Results

The number of AH patients in the offspring generation (to whom risks were calculated) was 3,274 (43.0% male) with a mean diagnostic age (i.e., first hospital contact) of 34.2 years for men and 38.8 years for women; considering also their parents the total number was 6,269, of whom 42.3.0% were males (Table 1). The respective patient numbers for PBC were 1.695 (17.8% male) and 4,269, with 27.0% males. The total AID population amounted to 519,180 patients in the offspring generation of 8.5 million. Thus AH accounted for 0.6% and PBC for 0.3% of all AIDs in the offspring generation, and 0.8% and 0.6% in the whole population. Demographic data on the offspring population are shown in the S1 Table.

**Table 1:** Number of cases of autoimmune diseases in offspring and in the total population, 1964–2012.

	No. of events in the offspring population			No. of events in the total of population	
	No.	%	Mean age	No.	% of events in the offspring population to the total of population
All autoimmune diseases	519,180		38.8 ± 19.5	769,991	67.4
Males	229,975	44.3	38.5 ± 19.5	309,359	74.3
Females	289,205	55.7	39.1 ± 19.4	460,632	62.8
**Subtype**					
Autoimmune hepatitis	3274	0.6	36.8 ± 16.6	6269	52.2
Males	1407	0.6	34.2 ± 14.9	2652	53.1
Females	1867	0.6	38.8 ± 17.5	3617	51.6
Primary biliary cholangitis	1695	0.3	53.1 ± 14.0	4269	39.7
Males	301	0.1	48.7 ± 19.1	1149	26.2
Females	1394	0.5	54.0 ± 12.4	3120	44.7

### Concordant familial risks

Familial risks for the liver AIDs are shown in Table 2 for offspring whose first-degree relatives (parents or siblings as probands) were diagnosed with concordant disease. For AH only the risk between siblings was significant (3.83). For PBC the risks for offspring (9.05) and siblings (10.88) were almost equally high. Spousal risks were very high, 5.91 and 6.07 for AH: for PBC only one couple shared the risk. The 8 couples sharing diagnosis of AH were diagnosed at a mean age of 47 years (husbands) and 41 years (wives). The couples had combined 18 children; one of them was diagnosed with celiac disease in adult age and another from another family was diagnosed also with celiac disease shortly after birth.

**Table 2:** Familial risks of concordant autoimmune diseases among first-degree relatives (top) and among spouses (bottom).

	Parents only				Sibling only			
	O	SIR	95% CI		O	SIR	95% CI	
Autoimmune hepatitis	8	1.95	0.83	3.87	14	**3.83**	**2.09**	**6.45**
Primary biliary cholangitis	15	**9.05**	**5.05**	**14.97**	14	**10.88**	**5.93**	**18.30**
	Husbands				Wives			
	O	SIR	95% CI		O	SIR	95% CI	
Autoimmune hepatitis	8	**5.91**	**2.53**	**11.71**	8	**6.07**	**2.59**	**12.02**

Bold type: 95% CI does not include 1.00.

O = observed number of cases; SIR = standardized incidence ratio; CI = confidence interval

Sex specific familial risks are shown in Table 3 using any first-degree relatives as probands. For AH the risk for males and females were almost identical (3.18 and 3.14) but for PBC only the female SIR was significant (11.98).

**Table 3:** Familial risks of concordant liver autoimmune diseases.

	Both genders					Women				Men			
AID	**O**	**SIR**	P*	**95% CI**		**O**	**SIR**	**95% CI**		**O**	**SIR**	**95% CI**	
Autoimmune hepatitis	22	**3.16**	**3.2 x 10**^**−4**^	**1.98**	**4.79**	12	**3.14**	**1.61**	**5.50**	10	**3.18**	**1.51**	**5.87**
Primary biliary cholangitis	29	**10.72**	**3.8 x 10**^**−29**^	**7.12**	**15.52**	26	**11.98**	**7.82**	**17.57**	2	4.54	0.43	16.69

Bold type: 95% CI does not include 1.00.

O = observed number of cases; SIR = standardized incidence ratio; CI = confidence interval

* P values Bonferroni-corrected; 0.00 = <1.00 x 10^−180^

### Discordant familial risks

We analyzed familial risks for discordant AIDs and the results are shown in Table 4 when any of the bidirectional analyses were significant. AH risk was 2.21 (N = 11, 1.10–3.71) in families of PBC, and it was 2.47 (8, 1.05–4.48) for PBC in families of AH patients; these results are not repeated in Table 4. Among other AIDs, 14 showed a significant association with AH, and the SIRs were over 2.00 for discoid lupus erythematosus and immune thrombocytopenic purpura in families of AH patients, and for AH in families of polymyositis/dermatomyositis patients; of these only the last association was shared with PBC. Among other AIDs, 16 showed a significant association with PBC, and the SIRs were over 4.00 for systemic sclerosis in families of PBC patients, and for PBC in families of Behcet disease and pemphigus patients; case numbers for the latter two were low, but none of these three AIDs were associated with AH. For 10 AIDs one of the bidirectional associations were shared by AH and PBC. Sex-specific risks were also analyzed but none were significantly different from each other, and not shown.

**Table 4:** Familial risks for discordant AIDs.

		Both Genders				
Subtypes of AID in offspring	Family history of AID	O	SIR	95% CI		P
**Autoimmune hepatitis**	Celiac disease	25	1.44	0.93	2.06	0.07
Celiac disease	Autoimmune hepatitis	**69**	**1.68**	**1.31**	**2.10**	**0.00**
**Autoimmune hepatitis**	Crohn disease	**53**	**1.61**	**1.20**	**2.07**	**0.00**
Crohn disease	Autoimmune hepatitis	**65**	**1.27**	**0.98**	**1.60**	**0.05**
**Autoimmune hepatitis**	Diabetes mellitus type I	9	1.06	0.48	1.86	0.88
Diabetes mellitus type I	Autoimmune hepatitis	**49**	**1.43**	**1.05**	**1.85**	**0.01**
**Autoimmune hepatitis**	Discoid lupus erythematosus	7	1.82	0.72	3.43	0.13
Discoid lupus erythematosus	Autoimmune hepatitis	**11**	**2.77**	**1.37**	**4.65**	**0.00**
**Autoimmune hepatitis**	Graves	**68**	**1.27**	**0.98**	**1.59**	**0.05**
Graves	Autoimmune hepatitis	**74**	**1.30**	**1.02**	**1.61**	**0.02**
**Autoimmune hepatitis**	Immune thrombocytopenic purpura	12	1.39	0.72	2.29	0.27
Immune thrombocytopenic purpura	Autoimmune hepatitis	**24**	**2.03**	**1.30**	**2.93**	**0.00**
**Autoimmune hepatitis**	Multiple sclerosis	**41**	**1.61**	**1.15**	**2.14**	**0.00**
Multiple sclerosis	Autoimmune hepatitis	**50**	**1.32**	**0.98**	**1.71**	**0.05**
**Autoimmune hepatitis**	Polymyalgia rheumatica	**70**	**1.60**	**1.24**	**1.99**	**0.00**
Polymyalgia rheumatica	Autoimmune hepatitis	17	1.23	0.72	1.89	0.40
**Autoimmune hepatitis**	Polymyositis/dermatomyositis	**7**	**2.24**	**0.89**	**4.20**	**0.04**
Polymyositis/dermatomyositis	Autoimmune hepatitis	5	1.89	0.60	3.92	0.18
**Autoimmune hepatitis**	Primary biliary cholangitis	**11**	**2.21**	**1.10**	**3.71**	**0.01**
Primary biliary cholangitis	Autoimmune hepatitis	**8**	**2.47**	**1.05**	**4.48**	**0.01**
**Autoimmune hepatitis**	Psoriasis	134	1.09	0.91	1.28	0.34
Psoriasis	Autoimmune hepatitis	**202**	**1.21**	**1.05**	**1.38**	**0.01**
**Autoimmune hepatitis**	Rheumatoid arthritis	**189**	**1.43**	**1.23**	**1.64**	**0.00**
Rheumatoid arthritis	Autoimmune hepatitis	**152**	**1.79**	**1.52**	**2.09**	**0.00**
**Autoimmune hepatitis**	Sarcoidosis	**33**	**1.47**	**1.01**	**2.02**	**0.03**
Sarcoidosis	Autoimmune hepatitis	34	1.24	0.86	1.69	0.21
**Autoimmune hepatitis**	Systemic lupus erythematosus	13	1.33	0.71	2.15	0.32
Systemic lupus erythematosus	Autoimmune hepatitis	**17**	**1.76**	**1.02**	**2.69**	**0.02**
**Autoimmune hepatitis**	Ulcerative colitis	**82**	**1.43**	**1.14**	**1.76**	**0.00**
Ulcerative colitis	Autoimmune hepatitis	**134**	**1.55**	**1.30**	**1.82**	**0.00**
**Primary biliary cholangitis**	Behcet disease	**2**	**8.02**	**0.76**	**22.98**	**0.02**
Behcet disease	Primary biliary cholangitis	2	3.05	0.29	8.75	0.20
**Primary biliary cholangitis**	Celiac disease	**15**	**2.04**	**1.14**	**3.20**	**0.01**
Celiac disease	Primary biliary cholangitis	**43**	**1.91**	**1.38**	**2.53**	**0.00**
**Primary biliary cholangitis**	Crohn disease	16	1.08	0.62	1.68	0.77
Crohn disease	Primary biliary cholangitis	**63**	**1.80**	**1.38**	**2.27**	**0.00**
**Primary biliary cholangitis**	Diabetes mellitus type I	4	1.77	0.46	3.94	0.30
Diabetes mellitus type I	Primary biliary cholangitis	**35**	**1.93**	**1.34**	**2.62**	**0.00**
**Primary biliary cholangitis**	Giant-cell arteritis	11	1.30	0.64	2.18	0.41
Giant-cell arteritis	Primary biliary cholangitis	**15**	**2.01**	**1.12**	**3.16**	**0.01**
**Primary biliary cholangitis**	Glomerular nephritis chronic	11	1.37	0.68	2.30	0.31
Glomerular nephritis chronic	Primary biliary cholangitis	**22**	**1.80**	**1.12**	**2.63**	**0.01**
**Primary biliary cholangitis**	Graves	33	1.27	0.87	1.74	0.17
Graves	Primary biliary cholangitis	**56**	**1.36**	**1.03**	**1.74**	**0.02**
**Primary biliary cholangitis**	Hashimoto thyroiditis	**25**	**1.63**	**1.05**	**2.33**	**0.02**
Hashimoto thyroiditis	Primary biliary cholangitis	36	1.20	0.84	1.62	0.29
**Primary biliary cholangitis**	Pemphigus	**3**	**4.74**	**0.89**	**11.63**	**0.02**
Pemphigus	Primary biliary cholangitis	2	2.83	0.27	8.12	0.23
**Primary biliary cholangitis**	Polymyositis/dermatomyositis	**5**	**3.21**	**1.01**	**6.65**	**0.01**
Polymyositis/dermatomyositis	Primary biliary cholangitis	4	2.02	0.53	4.49	0.20
**Primary biliary cholangitis**	Psoriasis	**77**	**1.35**	**1.07**	**1.67**	**0.01**
Psoriasis	Primary biliary cholangitis	**161**	**1.34**	**1.14**	**1.55**	**0.00**
**Primary biliary cholangitis**	Rheumatoid arthritis	**97**	**1.37**	**1.11**	**1.66**	**0.00**
Rheumatoid arthritis	Primary biliary cholangitis	**92**	**1.39**	**1.12**	**1.69**	**0.00**
**Primary biliary cholangitis**	Sarcoidosis	**18**	**1.62**	**0.96**	**2.46**	**0.04**
Sarcoidosis	Primary biliary cholangitis	**39**	**1.91**	**1.36**	**2.55**	**0.00**
**Primary biliary cholangitis**	Systemic lupus erythematosus	9	1.86	0.84	3.27	0.07
Systemic lupus erythematosus	Primary biliary cholangitis	**18**	**2.53**	**1.50**	**3.83**	**0.00**
**Primary biliary cholangitis**	Systemic sclerosis	**6**	**2.97**	**1.07**	**5.82**	**0.01**
Systemic sclerosis	Primary biliary cholangitis	**10**	**4.04**	**1.92**	**6.93**	**0.00**
**Primary biliary cholangitis**	Ulcerative colitis	**38**	**1.43**	**1.01**	**1.92**	**0.03**
Ulcerative colitis	Primary biliary cholangitis	**89**	**1.47**	**1.18**	**1.79**	**0.00**

Bold type: 95% CI does not include 1.00.

O = observed number of cases; SIR = standardized incidence ratio; CI = confidence interval

### Summarizing concordant and discordant associations

**Fig 1:**
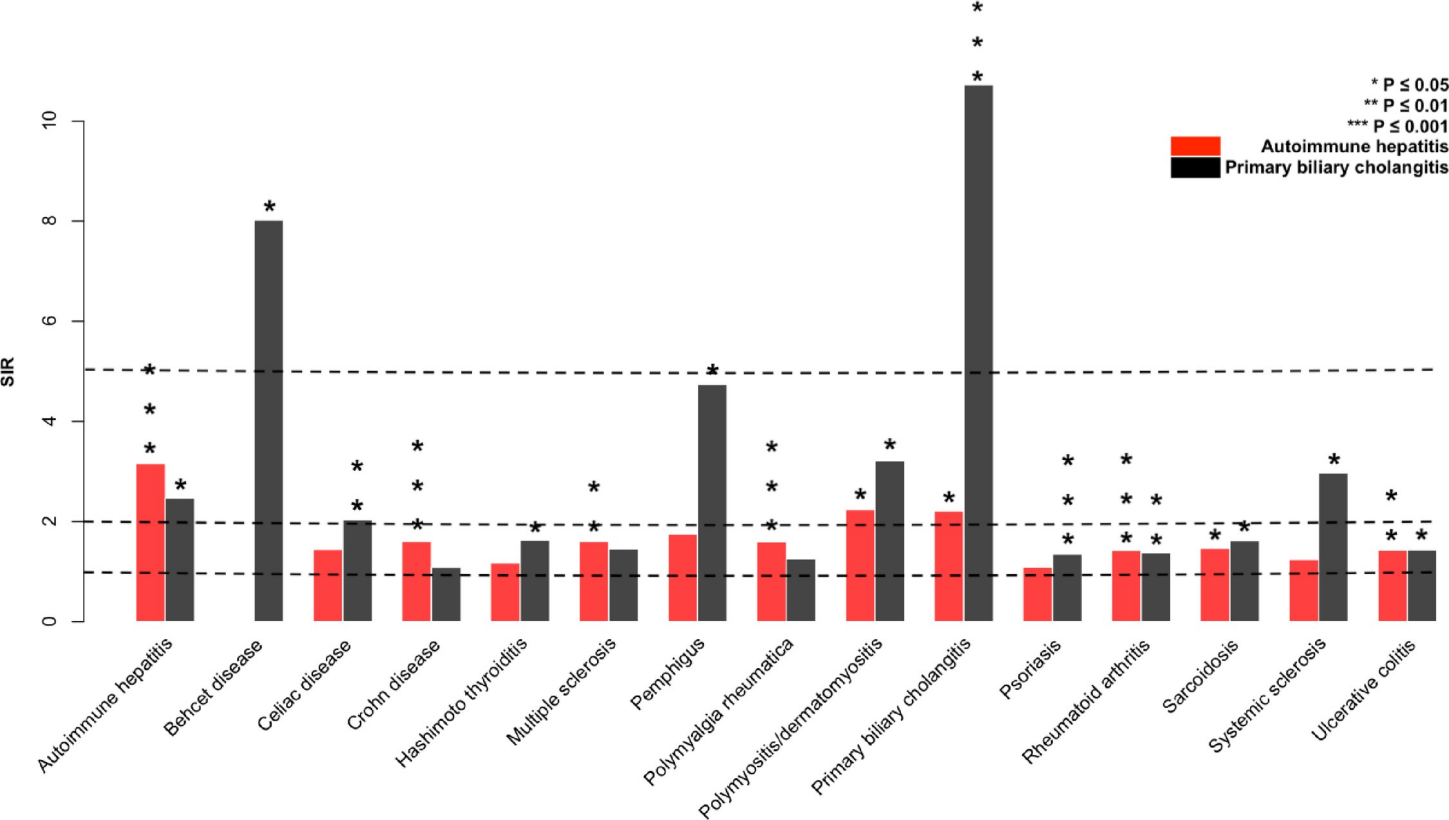
Familial associations of autoimmune hepatitis and primary biliary cholangitis with concordant and discordant AIDs. Families with autoimmune hepatitis had no Bechet disease patients. Statistically significant associations are shown by stars on top of the bars.

Familial risks are shown for both AH and PBC when at least one discordant risk in a family member was significant (Fig 1). AH was associated with 7 AIDs (not including PBC), of which Crohn disease, polymyalgia rheumatica and rheumatoid arthritis were the most significant. PBC was associated with 10 other AIDs of which psoriasis was the most significant. AH and PBC shared significant association with polymyositis/dermatomyositis, rheumatoid arthritis, sarcoidosis and ulcerative colitis.

## Discussion

The present nation-wide study covered two relatively rare AIDs affecting the liver; AH accounted for 0.8% of all hospitalized AIDs and PBC accounted for 0.6%. While previous literature is available on concordant familial risks for these AIDs, the present discordant familial risks are largely novel. A striking finding for AH showed extraordinary high concordance between spouses, with an SIR of 6.0. For AH there was no significant risk between offspring and parents, while the risks between siblings reached an SIR of 3.83, still less than the risk between spouses. The higher risk between siblings than between offspring and parents is in line with a higher environmental sharing between siblings. We have assessed the spouse correlation as a measure of environmental sharing in numerous family studies on AIDs, cancers and other diseases but never before found that spouse risk exceeds risk between offspring and parents [17–20]. The highest previous spouse correlation (2.35) was found for amyotrophic lateral sclerosis but this was still well below the risk between offspring and parents (4.71) [21]. We checked AIDs diagnoses in the 18 children of the couples affected by AH, and, curiously, two children from separate families were diagnosed with celiac disease. The result suggests that spouse correlation is the results of very strong environmental risk factors that overshadows any possible genetic associations between family members. However, genetic risk factors, including allelic variants in the HLA locus, have been associated with susceptibility and gene-environment interactions are thus likely [22]. Suggested environmental triggers include drugs and herbal agents, infections, alcohol, vitamin D deficiency, and an altered composition of the intestinal microbiome [22, 23]. Alcohol would be a potential candidate but the sex distribution and a small case-control study on AH do not support a role for it [24].

For PBC, the high concordant association of about 10 was in line with the population-based Icelandic data [15]. AH and PBC showed shared familial associations with each other, the SIRs being 2.21 and 2.47, which is in line with polyautoimmunity between these AIDs [5]. AH and PBC shared any significant bidirectional associations with 10 other AIDs, most of which were lower than those between AH and PBC, suggesting that the two liver AIDs share somewhat more with each other than with extrahepatic AIDs. Polymyositis/dermatomyositis showed some high SIRs for both AH and PBC but all the highest discordant association were specific to AH (discoid lupus erythematosus and immune thrombocytopenic purpura) or to PBC (systemic sclerosis, Behcet disease and pemphigus patients). Although the results provide evidence on familial polyautoimmunity for the liver AIDs, the magnitudes of discordant risks associated with PBC were very modest, compared with a high concordant risk of 10. The discordant SIRs were all well below 2.0, for the common AIDs, such as psoriasis, rheumatoid arthritis, and thyroid AIDs. While AH showed no significant association with common AIDs, such as celiac disease (in spite of two celiac diagnoses in 18 children of the spouses both diagnosed with AH), Hashimoto thyroiditis and psoriasis, it showed consistent associations with AIDs targeting the bowel, Crohn disease and ulcerative colitis.

Limitations of the present study on rare AIDs include low patient numbers, and many comparisons whereby some of the results are bound to yield chance findings. The design of applying bidirectional analysis will in part alleviate the problem of multiple comparisons because parent-offspring pairs are essentially independent in the bidirectional analysis. Another limitation is that we have no information on co-morbidities in the study population because only the first AID was considered for each person to simplify the family study. However, the previous Swedish studies on AH and PBC report such data and the population in the PBC study is overlapping with the present one [3, 5]. A further weakness was the definition of spouses which was based on the first common child and would not tell which of the couples subsequently divorced. Thus the risk estimate for spouses is likely to be an underestimate. One may also question how generalizable the present results are, as we included only hospitalized patients. However, in Sweden where medical care is practically free of charge, diagnostics and treatment at the specialist level are largely conduced at inpatient or outpatient wards in hospitals. The reported prevalence in PBC was among the highest in the world from the overlapping patient population, suggesting that the coverage of patients was high (5). The strengths of the study were diagnostics in a standard way in a high-level health care system, accessible to the population at large without economic barriers. The family relationships were obtained from the Swedish Multigeneration Register with unbiased and complete family relationships [25].

In summary, the present study assessed concordant and discordant familial relations for liver AIDs AH and PBC, which accounted for 0.8 and 0.6% of all hospitalized AIDs in Sweden. The surprising finding was the high risk (6.0) for spouses to be diagnosed with AH. The lower familial risk for AH between siblings (3.83) suggests that strong environmental and/or gene-environmental risk factors predispose to AH. PBC showed a high concordant familial risk of 10.0 and also was associated with AH. Both liver AIDs were moderately associated with multiple other AIDs, of which 10 were shared with AH and PBC. The results call attention to environmental factors in these complex diseases which should also be in focus in taking anamnestic data from patients.

## Supporting information

S1 Table (DOCX)
