# Supplementary material for: Familial associations between autoimmune hepatitis and primary biliary cholangitis and other autoimmune diseases
Source: PLoS One. 2020 Oct 20;15(10):e0240794. doi: 10.1371/journal.pone.0240794 (PMC7575086; doi:10.1371/journal.pone.0240794)
Supplement: S1 Table — (DOCX) [file pone.0240794.s001.docx]

| **S1 Table. Characteristics of study population and patients in offspring** | | | | | | | | |
| --- | --- | --- | --- | --- | --- | --- | --- | --- |
|  | Population | |  | Autoimmune hepatitis | |  | Primary biliary cholangitis | |
|  | No. | % |  | No. | % |  | No. | % |
| **Total** | 8517416 |  |  | 3274 |  |  | 1695 |  |
| **Gender** |  |  |  |  |  |  |  |  |
| Males | 4367206 | 51.3 |  | 1407 | 43.0 |  | 301 | 17.8 |
| Females | 4150210 | 48.7 |  | 1867 | 57.0 |  | 1394 | 82.2 |
| **Age at diagnosis (years)** |  |  |  |  |  |  |  |  |
| < 20 |  |  |  | 477 | 14.6 |  | 66 | 3.9 |
| 20-29 |  |  |  | 771 | 23.5 |  | 36 | 2.1 |
| 30-39 |  |  |  | 692 | 21.1 |  | 101 | 6.0 |
| 40-49 |  |  |  | 568 | 17.3 |  | 319 | 18.8 |
| 50-59 |  |  |  | 391 | 11.9 |  | 596 | 35.2 |
| 60-69 |  |  |  | 268 | 8.2 |  | 470 | 27.7 |
| ≥ 70 |  |  |  | 107 | 3.3 |  | 107 | 6.3 |
| **Socioeconomic status** |  |  |  |  |  |  |  |  |
| Farmers | 98773 | 1.2 |  | 39 | 1.2 |  | 14 | 0.8 |
| Self-employed | 304141 | 3.6 |  | 124 | 3.8 |  | 63 | 3.7 |
| Professionals | 792527 | 9.3 |  | 300 | 9.2 |  | 132 | 7.8 |
| White collar workers | 1981334 | 23.3 |  | 918 | 28.0 |  | 600 | 35.4 |
| Blue collar workers | 3163458 | 37.1 |  | 1235 | 37.7 |  | 652 | 38.5 |
| Others | 2177183 | 25.6 |  | 658 | 20.1 |  | 234 | 13.8 |
| **Residential area** |  |  |  |  |  |  |  |  |
| Large cities | 2926239 | 34.4 |  | 1410 | 43.1 |  | 598 | 35.3 |
| Southern Sweden | 3705053 | 43.5 |  | 1384 | 42.3 |  | 803 | 47.4 |
| Northern Sweden | 1886124 | 22.1 |  | 480 | 14.7 |  | 294 | 17.3 |
